# The impact of untreated hearing loss on depression, anxiety, stress, and loneliness in tonal language-speaking older adults in China

**DOI:** 10.3389/fpsyg.2022.917276

**Published:** 2022-12-01

**Authors:** Xinxing Fu, Robert H. Eikelboom, Bo Liu, Shuo Wang, Dona M. P. Jayakody

**Affiliations:** ^1^Beijing Institute of Otolaryngology, Otolaryngology-Head and Neck Surgery, Beijing Tongren Hospital, Capital Medical University, Beijing, China; ^2^Medical School, The University of Western Australia, Crawley, WA, Australia; ^3^Ear Science Institute Australia, Subiaco, WA, Australia; ^4^Department of Speech-Language Pathology and Audiology, University of Pretoria, Pretoria, South Africa; ^5^Curtin Medical School, Curtin University, Bentley, WA, Australia; ^6^WA Centre for Health and Aging, The University of Western Australia, Crawley, WA, Australia

**Keywords:** depression, anxiety, stress, age-related hearing loss, mental health, loneliness, tonal language

## Abstract

**Background:**

Age-related hearing loss, mental health conditions, and loneliness commonly affect older adults. This study aimed to determine whether untreated hearing loss is independently associated with depression, anxiety, stress, and loneliness in tonal language-speaking older adults in China.

**Study design:**

Observational, cross-sectional study.

**Methods:**

293 older adults (111 males, *M* = 70.33 ± 4.90 years; 182 females, *M* = 69.02 ± 4.08 years) were recruited. All participants completed a pure tone audiometric hearing assessment, and provided information on living arrangements, marital status, leisure activities, tobacco and alcohol use, and medical history. The Mandarin version of the De Jong Gierveld Loneliness Scale was used to measure loneliness, and the Mandarin version of the 21-item Depression Anxiety Stress Scale (DASS-21) was used to assess a range of symptoms common to depression, stress, and anxiety of the participants. The analysis focused on determining the predictors of depression, anxiety, and stress, and the predictors of measures of loneliness.

**Results:**

Multiple stepwise regression analyses revealed that the emotional loneliness (*β* = 0.303*, p* < 0.001) and living status (*β* = 0.110, *p* = 0.048) significantly predicted DASS depression scores; emotional loneliness (*β* = 0.276, *p* < 0.001) and a history of vascular disease (*β* = 0.148, *p* = 0.009) were significantly related to DASS anxiety scores; emotional loneliness (*β* = 0.341, *p* < 0.001) and a history of vascular disease (*β* = 0.129, *p* = 0.019) significantly predicted DASS stress scores. Furthermore, multiple stepwise regression analyses showed that DASS stress scores (*β* = 0.333, *p* < 0.001), education years (*β* = −0.126, *p* = 0.020), marriage status (*β* = 0.122, *p* = 0.024), and a history of vascular disease (*β* = 0.111, *p* = 0.044) significantly predicted emotional loneliness; four-frequency average hearing loss (*β* = 0.149, *p* = 0.010) and DASS stress scores (*β* = 0.123, *p* = 0.034) significantly predicted social loneliness scale; and four-frequency average hearing loss (*β* = 0.167, *p* = 0.003) and DASS stress scores (*β* = 0.279, *p* < 0.001) also significantly predicted overall loneliness. There were no significant associations with high-frequency hearing loss.

**Conclusion:**

This study revealed that loneliness has a significant relationship both with hearing loss and aspects of mental health in an older adult Mandarin-speaking population. However, mental health was not significantly associated with hearing loss in this population.

## Introduction

The increasing human lifespan has resulted in a growing number of older people with health problems associated with aging. According to World Health Organization (WHO), in 2019, the number of people aged 65 years and above was estimated to be 703 million, which is expected to double in 2050. Most of the estimated increase in population is expected to occur in Eastern and South-Eastern Asia (United Nations, 2017). China has the world’s largest elderly population. The newly released results of China’s seventh population census show that China’s 2020 population stood at 1.41 billion of which 264 million people were aged 60 years and older, comprising 18.7% of the total population (Guo et al., 2019).

Worldwide more than 1.5 billion people experience some degree of hearing loss, equivalent to 20% of the global population, of whom at least 430 million have a hearing loss of moderate or higher severity in the better ear (Chadha et al., 2021). For older adults, the global prevalence of moderate or higher grades of hearing loss increases with age, rising from 12.7% at the age of 60 years to over 58.6% at 90 years (World Health Organization, 2021). Notable is that over 58% of the world’s population with moderate or higher-grade hearing loss is experienced by those above the age of 60 years (World Health Organization, 2021). When left unaddressed, hearing loss impacts many aspects of life, including listening and communication (Barker et al., 2017), social isolation and loneliness (Dawes et al., 2015; Shukla et al., 2020), and mental health (Jayakody et al., 2018a,b; Harithasan et al., 2020).

It is estimated that 4.4% of the global population suffers from depressive disorders (Friedrich, 2017), and 3.6% from anxiety disorders (Nochaiwong et al., 2021). The prevalence of depression and anxiety increases with older age, approximately 15% of older adults experience clinically significant depressive symptoms (Blazer, 2003), and 1–5% live with a major depressive disorder (Hasin et al., 2005). Depression in older adults is associated with emotional suffering, morbidity, higher risk of suicide, and mortality from other causes, and is a major societal financial burden (Vieira et al., 2014). Currently, mental and neurological disorders account for 7% of the total burden of disease among those aged over 70 years in mainland China, followed closely by cardiovascular disease, cancer, and chronic respiratory disease (Lu et al., 2019).

Recent evidence suggests that there is a relationship between hearing loss and depression in non-tonal language speakers in Western countries (Keidser and Seeto, 2017; Lawrence et al., 2020). Previous work has also established that there is an association between hearing impairment and anxiety (Cosh et al., 2018; Jayakody et al., 2018a). However, the nature of the mechanism that may underlie this association remains unclear. Furthermore, little is known whether similar findings can be observed in other cultural and linguistic settings, such as in China. On the surface, both aspects require further investigation.

Loneliness is a subjective negative feeling associated with perceived limitations in a wider social network (social loneliness) or the absence of a specific desired companion or relationship (emotional loneliness) (Fierloos et al., 2021). Approximately, one-third of adults over 60  years will experience loneliness (Grenade and Boldy, 2008). Loneliness adversely affects general health and well-being, and is associated with increased risk of mortality (Fierloos et al., 2021). Listening and communication difficulties negatively impact older adults’ social networks and can increase loneliness (Cohen-Mansfield et al., 2016). Thus, loneliness may be exacerbated by increasing hearing loss.

Although loneliness is experienced by people across the world regardless of culture, gender, and age, there are substantial differences across cultures in the ways loneliness is experienced and coped with (van Staden and Coetzee, 2010). For example, in African populations, a lack of social connectivity is a major factor in loneliness; In Canadian culture, loneliness is more positively evaluated. Experiences of loneliness among different cultures may also vary because of unique expectations regarding relationships. There is evidence showing that loneliness is more about personal expectations in an individualist culture, whereas it is more about social approval in a communal culture (Yum, 2003). A study conducted in five European countries showed that higher collectivism was related to lower loneliness (Heu et al., 2019).

Traditional and contemporary Chinese culture places great importance on family members’ dependence, marital mutual support, and filial piety of offspring (Yang and Gu, 2021). With the enormous socioeconomic changes in China, increasing demand for childcare has resulted in greater availability of grandparents and reliance on them. More than half of grandparents in China provide care for their grandchildren (Xu, 2019). There is evidence showing that the grandparental role is a protective factor for both elderly loneliness (Zhang et al., 2021) and mental health issues (Xu, 2019). Whether the Chinese culture is protective of loneliness for those with hearing loss has not been established.

Mandarin is the dominant dialect of what is commonly known as Chinese, and is considered the standard language in China. The Chinese (or Sinitic) family of dialects is tonal in nature. Tonal languages employ lexical tones or pitch variations to indicate different meanings at the suprasegmental level (Yip, 2002). The number of tones in Mandarin is four, and nine in Cantonese, the next most common Chinese dialect. Regardless, tonal language speech perception requires the ability to discriminate subtle changes in tones. As these changes cannot be observed through articulatory movements, listeners have to rely on their hearing ability (Han et al., 2020). Therefore, hearing loss in tonal language speakers and listeners could have a significant impact on speech perception and therefore the ability to communicate with others. Consequently, hearing loss in the Chinese population may have a different relationship with aspects of mental health and loneliness than it does in non-tonal speaking populations.

As it is the case for the implications of culture on the relationship between hearing loss and aspects of mental health and loneliness, the influence of tonal language on these associations has not been properly explored yet. A cross-sectional study investigated the impact of hearing loss on loneliness in older people in China, showing that living arrangements had a significant modifying effect on loneliness (Jiang et al., 2021). Having offspring in the same community may play a protective role in reducing the loneliness of older adults with hearing loss (Jiang et al., 2021).

The nature of tonal languages also suggests further investigation is required into hearing sensitivity in the different bands of frequencies. For the most part, studies in non-tonal language settings that have investigated the association between hearing loss and mental health in older adults, have employed the average of the pure-tone hearing thresholds between 0.5 and 4 kHz (speech-frequency) or even self-reported hearing impairment (Lawrence et al., 2020) as measures of hearing loss. However, there is a substantial difference between Chinese and English (as an example of a non-tonal language) on the band information function (BIF), which quantify the contributions among frequency regions (Chen et al., 2016). Similar to other tonal languages, in Chinese the same syllable can convey different meanings by using different tones. The perception of tone primarily depends on the fundamental frequency (F0) contour. On the other hand, in English, the F0 only impacts the intonation, which will not change the meaning of the word (Du et al., 2019). Furthermore, high frequencies (6–8 kHz) show a decrease in sensitivity at least a decade earlier than for the speech frequencies (Salvi et al., 2018), and hearing ability at these frequencies impacts speech perception (Feng et al., 2010). Chinese Mandarin speakers may be less susceptible to high-frequency hearing loss than non-tonal language speakers as they age. Therefore, the association between hearing loss in speech and high frequencies and mental health and loneliness should be explored.

The purpose of this study was to determine whether untreated hearing loss is independently associated with depression, anxiety, stress (collectively referred to in report as mental health), and loneliness in Mandarin-speaking older adults in China.

## Materials and methods

The protocols for this study have previously been reported for a study on hearing loss and cognitive function in an adult Chinese population (Fu et al., 2021). Participants were recruited at the Beijing Institute of Otolaryngology, through social media advertisements, flyers, and community events. Ethics approval for this study was obtained from The University of Western Australia-Human Research Ethics Committee (RA/4/20/5538) and Beijing Tongren Hospital, Capital Medical University (TRECKY2019-090). All procedures were carried out in accordance with these approvals and the participants all provided written informed consent.

Native Mandarin speakers aged 60 years and above were invited to participate in the study. Those not in a general state of good health, or unable to perform tasks required in the mental health evaluation session due to an underlying physical or mental condition, or those who had previously worn or currently wearing hearing aids or a hearing implant were excluded from the study, or those with single-side deafness were also excluded, which refers to subjects with severe-to-profound hearing loss on one side and normal hearing on the other side.

After enrolment, all participants completed a baseline questionnaire capturing data on demographic characteristics (date of birth, sex, and years of education), alcohol consumption (never, less than 14, 15–28, 29–42, or 43 or more standard drinks per week) (Kerr and Stockwell, 2012), smoking (never, past, current, or exposed to second-hand smoking), self-reported chronic disease history (heart disease, stroke, high cholesterol, atherosclerosis, hypertension, diabetes, frequent childhood ear infections, trauma to ear or head, and depression), leisure activities, as well as marital status (single, married, widowed, or divorced), and living arrangements (living alone, living with a spouse, or living with children). Leisure activities were classified into recreational, intellectual, physical, and social categories (Leung et al., 2010).

The assessment of participants comprised measurements of hearing ability, loneliness, and mental health.

### Hearing assessment

Pure-tone audiometry (PTA) was assessed with an audiometer (Conera Audiometer, GN Otometrics Ltd., Denmark) and supra-aural earphone (TDH-39). For all participants, bilateral pure-tone air-conduction thresholds were measured at 0.25, 0.5, 1, 2, 4, 6, and 8 kHz; and bone-conduction thresholds were measured at 0.5, 1, 2, and 4 kHz, through a standard audiometric assessment conducted by a qualified audiologist in a soundproof booth at the audiology center of the Beijing Institute of Otolaryngology.

Hearing loss data were summarized by two different methods: four frequency average of hearing thresholds at 0.5, 1, 2, and 4 kHz (speech frequencies), and the three high-frequency average of hearing thresholds at 4, 6, and 8 kHz (high frequencies), noted, respectively, as 4FA and 3HFA. In both cases, the value for the better ear was used for further analysis, being a measure of bilateral hearing loss. These were analyzed as continuous variables. Furthermore, for the purpose of describing the hearing loss of the study cohort, the 4FA hearing loss was classified using the World Health Organization (WHO) grades of hearing impairment (Humes, 2019), respectively, normal hearing—less than 20 dB HL; mild hearing loss—20 to <35 dB HL; moderate hearing loss—35 to <50 dB HL; moderately severe hearing loss—50 to <65 dB HL; severe hearing loss—65 to <80 dB HL; profound hearing loss—80 to <95 dB HL; and complete hearing loss—95 dB HL or greater in the better ear.

### Loneliness measurement

The Mandarin version of the six-item De Jong Gierveld Loneliness Scale was used to measure the loneliness of the participants (Leung et al., 2008; Yang et al., 2018; Fung et al., 2019). In this six-item scale, three statements are made about emotional loneliness and three about social loneliness, and answered as Yes, More, or less, or No. A total loneliness score, and sub-scores for social and emotional loneliness are calculated, taking into account the negative or position phrasing of the item (Leung et al., 2008).

### Assessment of depression, anxiety, and stress

The Depression Anxiety Stress Scales (DASS-21) is a 21-item questionnaire used to assess the symptoms of depression, anxiety, and stress (Chan et al., 2012; Wang et al., 2016). A Chinese version of DASS-21 was validated taking into account cross-cultural factors (Wang et al., 2016; Jiang et al., 2020). Responses are provided on a four-point Likert scale (0: “Did not apply to me at all,” 1: “Applied to me to some degree or some of the time,” 2: “Applied to me to a considerable degree or a good part of the time,” and 3: “Applied to me very much or most of the time”). Depression, anxiety, and stress scores are determined by adding the scores of the related items (Lovibond and Lovibond, 1995). As the DASS-21 is a shorter version of the 42-item original DASS, the score for each subscale is multiplied by 2 to calculate the final score.

### Statistics analysis

All statistical analyses were performed using SPSS version 25 (SPSS Inc., Chicago, IL, United States). The Kruskal Wallis H test for non-normally distributed data was used to investigate whether DASS-21, and loneliness scores were significantly different across three groups of participants classified by the severity of hearing loss (normal hearing, mild, and moderate hearing loss and moderately severe or above; based on better ear 4PTA).

A multiple forward stepwise regression analysis was firstly used to examine the relationship among mental health-related variables (DASS depression, DASS anxiety, and DASS stress) as the dependent variables, and these independent variables: age, gender, chronic medical history, education, 4FA, 3HFA, self-reported hearing loss, living status, marital status, emotional loneliness, social loneliness, overall loneliness, and the four activity scores (recreational, intellectual, physical, and social). The collinearities of factors were examined for each stepwise regression analysis, by using the variance inflation factor (VIF). Univariate ANOVA was used to test each independent variable against each of the dependent variables. Only those with *p* < 0.1 were included in the multiple regression analyses.

Secondly, a similar multiple forward regression analysis was conducted to examine the relationship between loneliness and the other variables. Emotional loneliness, social loneliness, and overall loneliness scores were entered as dependent variables. For the independent variables, a univariate ANOVA was used to test each independent variable against the dependent variables, and only those with *p* < 0.1 were included in the multiple forward regression analysis. The candidate independent variables were age, gender, chronic medical history, clinically diagnosed depression, education, 4FA, 3HFA, self-reported hearing loss, living status, marital status, DASS depression, DASS anxiety, DASS stress, and the four activity scores (recreational, intellectual, physical, and social).

Multiple forward regression analysis was conducted to examine the relationship between mental health, loneliness, and the other independent variables, across the three classifications of hearing loss: normal, mild, and moderate, and moderately severe.

## Results

### Descriptive data

A total of 293 people aged between 60 and 87 years were included in the study. The study cohort consisted of 111 males (mean age = 70.33 ± 4.90 years) and 182 females (mean age = 69.02 ± 4.08 years). A summary of the demographic information, classifications of hearing, mental health scores, loneliness scales, and other descriptive data of this study population are presented in Table 1.

**Table 1:** Participant’s descriptive data.

Characteristics	All subjects
	*n* = 293
Male Sex, No. (%)	111 (37.9%)
Smoking, No. (%)	107 (36.5%)
Alcohol consumption, No. (%)	279 (95.2%)
Vascular disease, No. (%)	231 (78.8%)
Diabetes, No. (%)	66 (22.5%)
Frequent childhood ear infections, No. (%)	13 (4.4%)
Depression clinically diagnosed, No. (%)	10 (3.4)
Self-report HL, No. (%)	212 (72.4%)
Noise Exposure, No. (%)	52 (17.7%)
Living alone, No. (%)	17 (5.8%)
Single or divorced, No. (%)	33 (11.3%)
Age (years)	69.52 ± 4.45
Education (years)	12.98 ± 2.84
Hearing loss classification, No. (%)	
Normal hearing	65 (22.2%)
Mild hearing loss	106 (36.2%)
Moderate hearing loss	54 (18.4%)
Moderately severe hearing loss	34 (11.6%)
Severe hearing loss	31 (10.6%)
Profound hearing loss	3 (1.0%)
4FA (dB HL)	36.94 ± 18.78
3HFA (dB HL)	52.31 ± 20.17
Emotional Loneliness	0.92 ± 0.84
Social Loneliness	1.11 ± 1.18
Overall Loneliness	2.03 ± 1.59
Depression	3.65 ± 5.22
Anxiety	5.98 ± 5.58
Stress	6.45 ± 6.70
Recreational-Activities Score	14.5 ± 10.68
Intellectual-Activities Score	20.87 ± 12.72
Physical-Activities Score	21.15 ± 11.06
Social-Activities Score	2.11 ± 2.79

The data are presented as means ± SDs, unless otherwise indicated. 4FA: four frequencies (500 Hz and 1, 2, and 4 kHz) average of pure tone hearing thresholds of the better ear; 3HFA: three high frequencies (4, 6, and 8 kHz) average of pure tone hearing thresholds of the better ear.

Alcohol consumption reference was classified as never, or less than 14 standard drinks per week.

Vascular disease indicates any one of heart disease, stroke, high cholesterol, atherosclerosis, or hypertension.

Group comparison of mental health and loneliness based on hearing loss: the median scores of mental health (anxiety, stress, and depression), and social and overall loneliness were significantly different (*p* < 0.05) between the three hearing loss groups, but not between emotional loneliness and hearing loss (see Supplementary material, Supplementary Table S-1).

### Regression analysis

Univariate ANOVA indicated for the following variables to be included in the multiple stepwise regression analysis; (i) For DASS depression: emotional loneliness, overall loneliness, history of vascular disease, and living status, (ii) for DASS anxiety: emotional loneliness, overall loneliness, and history of vascular disease, (iii) for DASS stress: emotional loneliness, social loneliness, overall loneliness, and history of vascular disease, (iv) for emotional loneliness: DASS depression, DASS anxiety, DASS stress, education years, marital status, history of vascular disease, 4FA, recreational activities, and history of mental health, (v) for social loneliness, DASS depression, DASS stress, 4FA, 3HFA, recreational activities, and social activities, and (vi) for overall loneliness, DASS depression, DASS anxiety, DASS stress, 4FA, 3HFA, history of vascular disease, and the social activities. All other independent variables were not significantly associated (*p* > 0.1).

All the tolerances and variance inflation factor (VIF) values met the prerequisites for the multiple regression analysis, to exclude collinearity between predictors. Multiple stepwise regression analysis revealed the following significant predictor of the dependent variables (see Table 2): (i) For DASS depression: emotional loneliness and living status, (ii) for DASS anxiety: emotional loneliness and history of vascular disease, (iii) for DASS stress: emotional loneliness and history of vascular disease, (iv) for emotional loneliness: DASS stress, education, marriage status and history of vascular disease, (v) for social loneliness: 4FA and the DASS stress, and (vi) for overall loneliness: 4FA and the DASS stress.

**Table 2:** Multiple stepwise regression between mental health/loneliness scores and other variables.

Dependent variable	Independent variables	*R* ^2^	Adjusted *R*^2^	*B*	*β*	*t*	Sig.	95% CI for B	
								Lower bound	Upper bound
Depression	Emotional loneliness	0.104	0.098	1.879	0.303	5.448	0.000	1.200	2.557
	Living status			2.460	0.110	1.987	0.048	0.024	4.897
Anxiety	Emotional loneliness	0.112	0.106	1.834	0.276	4.927	0.000	1.102	2.567
	Vascular disease			2.020	0.148	2.641	0.009	0.514	3.526
Stress	Emotional loneliness	0.148	0.142	2.720	0.341	6.213	0.000	1.859	3.582
	Vascular disease			2.117	0.129	2.353	0.019	0.346	3.887
Overall loneliness	Stress	0.111	0.105	0.066	0.279	5.033	0.000	0.040	0.092
	4FA			0.014	0.167	3.018	0.003	0.005	0.023
Emotional loneliness	Stress	0.171	0.159	0.042	0.333	6.078	0.000	0.028	0.055
	Education years			−0.037	−0.126	−2.331	0.020	−0.069	−0.006
	Marital status			0.324	0.122	2.274	0.024	0.044	0.605
	Vascular disease			0.227	0.111	2.021	0.044	0.006	0.449
Social loneliness	4FA	0.039	0.033	0.009	0.149	2.594	0.010	0.002	0.016
	Stress			0.022	0.123	2.131	0.034	0.002	0.041

4FA: four frequencies (500 Hz and 1, 2, and 4 kHz) average of pure tone hearing thresholds of the better ear.

Only the last model of the stepwise regression is shown in Table 2. Each model of forward stepwise regression for DASS depression, DASS anxiety, DASS stress, emotional loneliness, social loneliness, and loneliness are provided as Supplementary material, Supplementary Tables S-2–S-7.

Regression analysis using the hearing loss classified by severity rather than hearing loss as a continuous variable showed similar results to the above, except that 4FA was not significantly related to loneliness scores in normal hearing and moderately severe or above hearing loss group (see Supplementary Tables S-8–S-10).

## Discussion

This study investigated the association between untreated hearing loss and loneliness and mental health in Mandarin speaking older adults in China. To the best of our knowledge, this is the first study to examine the association between hearing loss, loneliness, and mental health by employing both speech-frequency and high-frequency audiometry in a Chinese-setting population.

Our results revealed that whether speech or higher frequency losses were considered, mental health scores, including depression, anxiety, and stress were not significantly associated with hearing loss. Our findings in this tonal language population do not align with those of cross-sectional studies of pure-tone audiometric hearing loss and mental health in non-tonal language speakers (Jayakody et al., 2018a; Marques et al., 2021; Tsimpida et al., 2022). These results may be explained by the fact that older adults in China may accept hearing loss as part of the typical aging experience. This is a quite common attitude to sensory loss in many Asian countries (Ji et al., 2015), where individuals are known to adapt to changes in their hearing by modifying their communication skills to reduce the encumbrance of hearing loss. These behavior changes help to navigate the potential negative impact of hearing loss on social isolation and loneliness and consequently reduce the potential effect on mental health (Cosh et al., 2018). This finding is consistent with another study conducted in Malaysia which found no association between sensory impairment and depression (Harithasan et al., 2020). The mean hearing threshold in the better ear in this current study was 36.94 dB HL. Therefore, whether more severe levels of hearing loss contribute to clinically significant depression, anxiety, or stress levels needs to be explored.

Sex, education level, and marital status were not significantly associated with mental health in these Chinese participants. These findings are partially corroborated by a previous study in which gender, marital status, and education are not related to depression in a Chinese oldest-old population, although the mean age of the subjects in this study was higher (Chou and Chi, 2005).

The loneliness scores from the present study were slightly higher than those reported by Leung et al. (2008) for the validated of the Chinese translation of the De Jong Gierveld Loneliness Scale in a group of elderly Hong Kong-based Chinese: 0.6 (SD 0.8) for emotional loneliness and 0.9 (SD 1.2) for social loneliness, compared to 0.92 (SD 0.84) and 1.11 (1.18), respectively for this study. A possible explanation for this may be that females report greater levels of loneliness than males (Pagan, 2020), and more female participants (62.1%) were enrolled in this study. Furthermore, the difference in age between the present study’s population and that of Leung et al. (2008) (Fung et al., 2019) may be another factor; a strong relationship between age and loneliness has been reported in the literature (Barreto et al., 2021). Loneliness levels in those over 50 years of age are characterized by a peak around 60 years of age and a dip around 75 years of age (Chou and Chi, 2005). The mean age of the participants in this study was 69.5 (SD 4.5) years, indicating that they may be closer to the peak of loneliness, compared to those in the study of Leung et al. where the mean age was 74.5 (SD 6.5) years.

All three mental health scales were significantly associated with emotional loneliness, but not social loneliness (Table 2). This association also accords with an earlier observation (Peerenboom et al., 2015), which showed that depression is strongly associated with the emotional dimension but not with the social dimension in a cohort study that investigated persons aged 60 years and above. A possible explanation for these results may be related to the difference between emotional and social loneliness. Emotional loneliness refers to a lack of other people with whom the individual can form an emotional attachment, which thereby may be the cause of depression (Tiikkainen and Heikkinen, 2005).

Our results also demonstrated that social and overall loneliness was significantly associated with speech-frequency hearing sensitivity. This finding accord with other studies, which have shown that greater hearing impairment was associated with greater degrees of loneliness (Pronk et al., 2011; Sung et al., 2016; Jiang et al., 2021). Two of these studies adopted the UCLA loneliness scale (Sung et al., 2016; Jiang et al., 2021), but did not differentiate social and emotional loneliness. Both studies revealed that older adults with hearing loss had significantly higher levels of loneliness than their age-matched groups with normal hearing. Pronk et al. (2011) measured loneliness by using the same scale as used in this study, finding that both social and emotional loneliness were associated significantly with hearing impairment, but only for men and for those without hearing aids. These relationships may be partly explained by the fact that age-related hearing loss degrades peripheral auditory processing function, limiting the individual’s ability to comprehend verbal information and making daily conversations difficult (Shukla et al., 2020). Thereby it may further lead to avoidance of potentially embarrassing social occasions, especially those with non-family members. That may also explain why this study found that hearing loss was only related to social loneliness, but not emotional loneliness. This difference could be attributed to hearing impairment causing less impact on emotional function than on social function. It has previously been reported (Fu et al., 2021), using the same data set as used in the present study, that social loneliness is associated with cognitive decline. Given the fact that a number of epidemiological studies have shown evidence that hearing loss is an independent modifiable risk factor for accelerating cognitive decline (Taljaard et al., 2016; Lau et al., 2021), more attention should be given to understanding the inter-relationships between hearing loss, social loneliness and cognitive decline. The evidence of the role of culture in the relationship between hearing loss and loneliness is inconclusive. Only marital status was found to be associated with emotional isolation. This requires further investigation, where there is a deeper examination of cultural aspects, potentially representing both individualistic and collectivistic populations.

The analysis showed that only speech-frequency hearing was associated with loneliness, not with the high frequency. High frequencies decline at least a decade earlier than the speech frequencies (Salvi et al., 2018), which does have an impact on speech perception. Therefore, an association with loneliness could be expected. It is difficult to explain why this was not found in this study, but it may be because only speech frequencies substantially impact daily conversation, and lead to potential loneliness. Besides, there is evidence showing that the different acoustic properties of Chinese spoken language may play a role. During the pronunciation of Mandarin, the frequency and intensity of the tones vary, and the shape of the Mandarin speech spectrum may be different from those derived from a non-tonal language (Hu et al., 2019). The main speech sounds congregated in frequencies around 0.5 and 2 kHz (Nicholas et al., 2021). Tone perception, which is important in speech perception in a tonal language, primarily depends on the fundamental frequency contour and the mid frequency range. Altogether, these could explain that loneliness in Chinese speakers was not affected by early high-frequency hearing loss.

Stress was observed to be associated with both the two dimensions of loneliness and overall loneliness. In accordance with the present results, previous studies have demonstrated that stress is causally related to loneliness, and it is hypothesized that changes produced in the hypothalamic–pituitary-adrenocortical (HPA) axis is related to stress (Campagne, 2019).

### Clinical implications

This study contributes to the growing global body of evidence that loneliness was significantly associated with hearing loss and mental health in tonal language-speaking older adults in China. Given the high burden of loneliness and mental health problems, interventions to alleviate these problems for seniors may have significant public health impacts. Hearing may be a modifiable solution for loneliness. Both otolaryngologists and physicians should discuss the long-term benefits of hearing loss treatment when managing and counseling their patients.

### Limitations

One of the limitations of the current study is that results are based on cross-sectional analyses rather than longitudinal assessments of hearing, loneliness, and mental health over time. Furthermore, the study would have benefited with the inclusion of more physically and mentally healthy participants, even though they are much more difficult to recruit from an older adult population. The study had a gender imbalance and had a relatively small sample size. Another limitation is that there were relatively fewer participants with untreated severe-to-profound hearing loss. These, too, are difficult to recruit from an older adult population. A longitudinal large-scale study, with a gender-balanced cohort that included physical and mental health participants, and a suitable representation of those with untreated severe to profound hearing loss, will help to examine a potential causal relationship between hearing loss, loneliness and mental health scores over time.

## Conclusion

Our research provides more evidence for the association between loneliness and hearing loss, and also between loneliness and mental health in Mandarin-speaking older adults in China. This study revealed that loneliness has a significant relationship with hearing loss and mental health in a Mandarin-speaking population.

## Data availability statement

The original contributions presented in the study are included in the article/Supplementary material, further inquiries can be directed to the corresponding authors.

## Ethics statement

The studies involving human participants were reviewed and approved by the University of Western Australia-Human Research Ethics Committee (RA/4/20/5538) and Beijing Tongren Hospital, Capital Medical University (TRECKY2019-090). The patients/participants provided their written informed consent to participate in this study.

## Author contributions

DJ and XF designed the experiments. XF carried out the experiments. XF, RE, DJ, SW, and BL analyzed the experimental data. XF wrote the manuscript. RE, DJ, SW, and BL reviewed the manuscript. All authors contributed to the article and approved the submitted version.

## Funding

This research was supported by the Reform and Development Grant of Beijing Institute of Otolaryngology, Natural Science Foundation of China (Grant No. 81200754), and National Key Research and Development Program (Grant No. 2020YFC2005200).

## Conflict of interest

The authors declare that the research was conducted in the absence of any commercial or financial relationships that could be construed as a potential conflict of interest.

## Publisher’s note

All claims expressed in this article are solely those of the authors and do not necessarily represent those of their affiliated organizations, or those of the publisher, the editors and the reviewers. Any product that may be evaluated in this article, or claim that may be made by its manufacturer, is not guaranteed or endorsed by the publisher.
