# Supplementary material for: The impact of untreated hearing loss on depression, anxiety, stress, and loneliness in tonal language-speaking older adults in China
Source: Front Psychol. 2022 Dec 1;13:917276. doi: 10.3389/fpsyg.2022.917276 (PMC9751871; doi:10.3389/fpsyg.2022.917276)
Supplement: Supplementary file 1 [file Data_Sheet_1.docx]

Supplementary material

The impact of hearing loss on mental health and loneliness in tonal language-speaking older adults in China

Xinxing Fu, Robert H. Eikelboom, Bo Liu, Shuo Wang, Dona M.P. Jayakody

Table S-1: Median and IQR scores of mental health (anxiety, stress and depression) and loneliness (emotional, social and overall) of participants according to the classification of severity of hearing impairment.

|  | NH N=65 | MMH N = 160 | MSPH N = 68 | Statistic χ2 | p-value |
| --- | --- | --- | --- | --- | --- |
| DASS Depression, median (IQR) | 2 (0, 6) | 0 (0, 4) | 4 (2, 6) | 14.446 | 0.001 |
| DASS Anxiety, median (IQR) | 6 (2, 10) | 4 (2,8) | 6 (4,8) | 9.823 | 0.007 |
| DASS Stress, median (IQR) | 6 (2, 10) | 4 (0,8) | 6 (4,10) | 9.869 | 0.007 |
| Emotion Loneliness, median (IQR) | 0 (0, 2) | 1 (0,1) | 1 (0, 2) | 5.379 | 0.068 |
| Social Loneliness, median (IQR) | 0 (0, 2) | 0 (0, 2) | 2 (0, 2) | 12.815 | 0.002 |
| Loneliness, median (IQR) | 1 (0, 3) | 2 (1, 3) | 2 (2, 4) | 14.369 | 0.001 |

NH: normal hearing—4FA less than 20 dB HL; MMH: 4FA—20 to < 50 dB HL; MSPH: 4FA—50 to < 95 dB HL.

IQR: interquartile range. χ2: chi-square.

Table S-2: Multiple stepwise regression between overall loneliness and other variables.

|  | Model 1 | | | | Model 2 | | | |
| --- | --- | --- | --- | --- | --- | --- | --- | --- |
| Variables | B | SE | β | Sig. | B | SE | β | Sig. |
| Stress | 0.068 | 0.013 | 0.288 | <0.001 | 0.066 | 0.013 | 0.279 | <0.000 |
| 4FA |  |  |  |  | 0.014 | 0.005 | 0.167 | 0.003 |
| R^2^ | 0.083 | | |  | 0.111 | | |  |
| Std. Error of the Estimate | 1.528 | | |  | 1.507 | | |  |

SE: Std. Error.

4FA: four frequencies (500, 1 kHz, 2 kHz and 4 kHz) average of pure tone hearing thresholds of the better ear.

Table S-3: Multiple stepwise regression between social loneliness and other variables.

|  | Model 1 | | | | Model 2 | | | |
| --- | --- | --- | --- | --- | --- | --- | --- | --- |
| Variables | B | SE | β | Sig. | B | SE | β | Sig. |
| 4FA | 0.010 | 0.004 | 0.156 | 0.008 | 0.009 | 0.004 | 0.149 | 0.010 |
| Stress |  |  |  |  | 0.022 | 0.010 | 0.123 | 0.034 |
| R^2^ | 0.024 | | |  | 0.039 | | |  |
| Std. Error of the Estimate | 1.163 | | |  | 1.156 | | |  |

SE: Std. Error.

4FA: four frequencies (500, 1 kHz, 2 kHz and 4 kHz) average of pure tone hearing thresholds of the better ear.

Table S-4: Multiple stepwise regression between emotional loneliness and other variables.

|  | Model 1 | | | | Model 2 | | | | Model 3 | | | | Model 4 | | | |  |
| --- | --- | --- | --- | --- | --- | --- | --- | --- | --- | --- | --- | --- | --- | --- | --- | --- | --- |
| Variables | B | SE | β | Sig. | B | SE | β | Sig. | B | SE | β | Sig. | B | SE | β | Sig. | |
| Stress | 0.045 | 0.007 | 0.362 | <0.001 | 0.045 | 0.007 | 0.356 | <0.001 | 0.042 | 0.007 | 0.333 | <0.001 | 0.042 | 0.007 | 0.333 | <0.001 | |
| Education Years |  |  |  |  | -0.034 | 0.016 | -0.116 | 0.034 | -0.037 | 0.016 | -0.126 | 0.029 | -0.037 | 0.016 | -0.126 | 0.020 | |
| Marital status |  |  |  |  |  |  |  |  | 0.324 | 0.143 | 0.122 | 0.029 | 0.324 | 0.143 | 0.122 | 0.024 | |
| Vascular disease |  |  |  |  |  |  |  |  |  |  |  |  | 0.227 | 0.112 | 0.111 | 0.044 | |
| R^2^ | 0.131 | | |  | 0.145 | | |  | 0.159 | | |  | 0.171 | | | |  |
| Std. Error of the Estimate | 0.785 | | |  | 0.780 | | |  | 0.775 | | |  | 0.771 | | | |  |

SE: Std. Error.

Table S-5: Multiple stepwise regression between depression and other variables.

|  | Model 1 | | | | Model 2 | | | |
| --- | --- | --- | --- | --- | --- | --- | --- | --- |
| Variables | B | SE | β | Sig. | B | SE | β | Sig. |
| Emotional loneliness | 1.182 | 0.347 | 0.303 | <0.001 | 1.879 | 0.345 | 0.303 | <0.000 |
| Living status |  |  |  |  | 2.460 | 1.238 | 0.110 | 0.048 |
| R^2^ | 0.092 | | |  | 0.104 | | |  |
| Std. Error of the Estimate | 4.979 | | |  | 4.954 | | |  |

SE: Std. Error.

Table S-6: Multiple stepwise regression between anxiety and other variables.

|  | Model 1 | | | | Model 2 | | | |
| --- | --- | --- | --- | --- | --- | --- | --- | --- |
| Variables | B | SE | β | Sig. | B | SE | β | Sig. |
| Emotional loneliness | 1.994 | 0.371 | 0.300 | <0.000 | 1.834 | 0.372 | 0.276 | <0.001 |
| Vascular disease |  |  |  |  | 2.020 | 0.765 | 0.148 | 0.009 |
| R^2^ | 0.090 | | |  | 0.112 | | |  |
| Std. Error of the Estimate | 5.331 | | |  | 5.277 | | |  |

SE: Std. Error.

Table S-7: Multiple stepwise regression between stress and other variables.

|  | Model 1 | | | | Model 2 | | | |
| --- | --- | --- | --- | --- | --- | --- | --- | --- |
| Variables | B | SE | β | Sig. | B | SE | β | Sig. |
| Emotional loneliness | 2.888 | 0.435 | 0.362 | <0.001 | 2.720 | 0.438 | 0.341 | <0.000 |
| Vascular disease |  |  |  |  | 2.117 | 0.900 | 0.129 | 0.019 |
| R^2^ | 0.131 | | |  | 0.148 | | |  |
| Std. Error of the Estimate | 6.253 | | |  | 6.205 | | |  |

SE: Std. Error.

S-8 Multiple stepwise regression between mental health/loneliness scores and other variables in normal hearing group.

| Dependent  Variable | Independent variables | R^2^ | Adjusted *R*^2^ | B | *β* | t | Sig. | 95% Confidence interval for B | |
| --- | --- | --- | --- | --- | --- | --- | --- | --- | --- |
|  |  |  |  |  |  |  |  | Lower Bound | Upper Bound |
| Depression | Living status | 0.122 | 0.108 | 8.393 | .349 | 2.959 | .004 | 2.725 | 14.061 |
| Anxiety | Emotional loneliness | 0.082 | 0.068 | 2.119 | .287 | 2.379 | .000 | .339 | 3.900 |
| Stress | Overall loneliness | 0.067 | 0.053 | .949 | .259 | 2.133 | .037 | .060 | 1.838 |
| Overall  Loneliness | Stress | 0.127 | 0.099 | .068 | .247 | 2.083 | .041 | .003 | .133 |
|  | Social activities |  |  | -.136 | -.245 | -2.058 | .044 | -.267 | -.0 |
| Emotional  Loneliness | Education Years | 0.219 | 0.180 | -.116 | -.387 | -3.355 | .001 | -.185 | -.046 |
|  | Marital status |  |  | .800 | .282 | 2.444 | .017 | .145 | 1.454 |
| Social  Loneliness | Social activities | 0.066 | 0.051 | -.100 | -.257 | -2.108 | .039 | -.194 | -.005 |

4FA: four frequencies (500, 1 kHz, 2 kHz and 4 kHz) average of pure tone hearing thresholds of the better ear.

S-9 Multiple stepwise regression between mental health/loneliness scores and other variables in mild and moderate hearing group.

| Dependent  Variable | Independent variables | R^2^ | Adjusted *R*^2^ | B | *β* | t | Sig. | 95% Confidence interval for B | |
| --- | --- | --- | --- | --- | --- | --- | --- | --- | --- |
|  |  |  |  |  |  |  |  | Lower Bound | Upper Bound |
| Depression | Emotional Loneliness | 0.114 | 0.110 | 2.013 | .337 | 5.383 | .000 | 1.276 | 2.749 |
| Anxiety | Emotional loneliness | 0.129 | 0.121 | 1.751 | .272 | 4.276 | .000 | .944 | 2.557 |
|  | Vascular disease |  |  | 2.377 | .184 | 2.895 | .004 | .759 | 3.996 |
| Stress | Emotional loneliness | 0.167 | 0.160 | 2.982 | .126 | 2.029 | .000 | 1.976 | 3.987 |
|  | Vascular disease |  |  | 2.078 | .126 | 2.029 | .044 | .060 | 4.096 |
| Overall  Loneliness | Stress | 0.108 | 0.100 | .066 | .288 | 4.562 | .000 | .037 | .094 |
|  | 4FA |  |  | .013 | .140 | 2.211 | .028 | .001 | .024 |
| Emotional  Loneliness | Stress | 0.169 | 0.162 | 0.044 | .362 | 5.842 | .000 | .029 | .059 |
|  | Vascular disease |  |  | .270 | .135 | 2.168 | .031 | .025 | .515 |
| Social  Loneliness | NA | - | - | - | - | - | - | - | - |

4FA: four frequencies (500, 1 kHz, 2 kHz and 4 kHz) average of pure tone hearing thresholds of the better ear.

S-10 Multiple stepwise regression between mental health/loneliness scores and other variables in moderately severe or above hearing group.

| Dependent  Variable | Independent variables | R^2^ | Adjusted *R*^2^ | B | *β* | t | Sig. | 95% Confidence interval for B | |
| --- | --- | --- | --- | --- | --- | --- | --- | --- | --- |
|  |  |  |  |  |  |  |  | Lower Bound | Upper Bound |
| Depression | Living status | 0.155 | 0.129 | 6.697 | .325 | 2.831 | .006 | 1.972 | 11.422 |
|  | Overall Loneliness |  |  | 0.621 | .266 | 2.313 | .024 | .085 | 1.158 |
| Anxiety | Emotional loneliness | 0.077 | 0.063 | 1.471 | .277 | 2.339 | .022 | .215 | 2.726 |
| Stress | Emotional loneliness | 0.118 | 0.104 | 1.972 | .343 | 2.966 | .004 | .645 | 3.299 |
| Overall  Loneliness | NA | - | - | - | - | - | - | - | - |
| Emotional  Loneliness | Stress | 0.118 | 0.104 | 0.060 | .343 | 2.966 | .004 | .020 | .100 |
| Social  Loneliness | NA | - | - | - | - | - | - | - | - |

4FA: four frequencies (500, 1 kHz, 2 kHz and 4 kHz) average of pure tone hearing thresholds of the better ear.
